# Topical Heparin in Burns: A Systematic Review and Meta-Analysis of Randomized Controlled Studies

**DOI:** 10.1093/jbcr/iraf168

**Published:** 2025-08-30

**Authors:** Moussa Nassar, Mohamed I Mohamed, Maryam Shahid, Rama Taha, Rashed W Alweshah, Marwa R Yousef, Yousra Eltagouri, Diaz G Gustavo

**Affiliations:** Gilbert and Rose-Marie Chagoury School of Medicine, Lebanese American University, Byblos, Lebanon; Alexandria University, Faculty of Medicine, Alexandria 21526, Egypt; Tbilisi State Medical University, 0186, Georgia; Mediclinic Airport Road Hospital, 48481, UAE; Alexandria University, Faculty of Medicine, Alexandria 21526, Egypt; Alexandria University, Faculty of Science, Alexandria 21568, Egypt; University of Sadat City, Faculty of Veterinary Medicine, Sadat City 32897, Egypt; Beth Israel Deaconess Medical Center, Boston, MA 02215, United States; Facultad de Medicina y Nutrición, Universidad Juarez del Estado de Durango, 34000, Mexico; Facultad de Medicina, Universidad Autónoma de Coahuila, 25000, Mexico

**Keywords:** heparin, topical heparin, burn, topically applied heparin, burns

## Abstract

Burns are associated with significant inflammation and pain. Topical agents like heparin can modulate these processes and improve outcomes. Our study aims to evaluate the effectiveness of using topical heparin (TH) in patients with burns. On August 7, 2024, we conducted a literature search on PubMed, Scopus, and Web of Science. Only randomized controlled studies were included. Data were extracted on analgesic drug usage, bleeding events, sepsis, visual analog scale pain scores, length of hospital stay, and mortality. Statistical analysis was performed using R software (version 4.4.1), heterogeneous data. Seven randomized controlled trials (503 patients; topical heparin: 249, control: 254) were included. Analgesic use (1–2 times/day: RR = 3.04, *P* = .68; 3–4 times/day: RR = 0.06, *P* = .18), bleeding (RR = 5.06, *P* = .37), sepsis (RR = 0.77, *P* = .40), hospital stay, and mortality (RR = 0.13, *P* = .90) showed no significant differences. Topical heparin reduced local wound infections by 60% (RR = 0.40, *P* < .01) and lowered Visual Analog Scale pain scores (MD = −3.34, *P* < .01). However, sensitivity analysis excluding an outlier nullified the pain reduction (MD = −4.17, *P* = .57). All studies had a high risk of bias, especially in outcome measurement and randomization. Topical heparin reduces pain and local wound infections in burn patients without having an impact on other outcomes. Evidence is limited by a high risk of bias. Well-designed randomized trials are needed to determine its broader clinical value.

## INTRODUCTION

Burns are injuries caused by various external factors, including heat, radiation, electricity, friction, and chemicals.^1^ Burn injuries remain a major global health concern, affecting millions of individuals annually and contributing to significant morbidity and healthcare costs.^2^ Effective burn management is crucial for minimizing complications, promoting wound healing, and improving patient outcomes. Standard treatment approaches include wound debridement, infection control, pain management, and surgical interventions when necessary. However, ongoing research continues to explore adjunct therapies that may enhance wound healing while reducing complications.^3^

Heparin, a widely used anticoagulant in thromboembolic disorders, has been investigated as a topical agent for burn management due to its potential anti-inflammatory, angiogenic, and wound-healing properties.^4^ Preclinical studies suggest that topical heparin (TH) may alleviate pain, modulate inflammatory responses, and enhance wound re-epithelialization in burn patients.^5^^,^^6^ Despite accumulating evidence, the efficacy and safety of TH in burn treatment remain debated, particularly concerning its effects on pain relief, complications, mortality, and hospital length of stay. Previous systematic reviews in this area are relatively outdated and suggest that heparin demonstrated potential benefits.^7^^,^^8^ Although previous literature has examined heparin in burn treatment, none have provided a focused and comprehensive analysis of the efficacy of topically applied heparin in burn wound healing. This meta-analysis represents the most up-to-date and comprehensive evaluation of this topic. This systematic review and meta-analysis aim to evaluate the therapeutic role of TH in burn management by analyzing randomized controlled trials.

## METHODS

This paper’s protocol is registered on PROSPERO using the following ID CRD42024578014 and follows the regulations of the preferred reporting items for systematic reviews and meta-analyses (PRISMA).^9^

### Search strategy

On August 7, 2024, a literature search was conducted across three databases: PubMed, Scopus, and Web of Science. The search strategy incorporated both keywords and MeSH headings, using terms such as: “Heparin” OR “heparin application” OR “topical heparin treatment” OR “heparin administration” AND “burn treatment” OR “burn wounds” OR “burn injuries” OR “burn management” OR “burn^*^.” Additionally, the reference lists of included studies and relevant previous reviews were manually screened to identify any potentially missed studies. The full search strategy is detailed in Supplementary File 1.

### Study selection

Randomized controlled trials (RCTs) evaluating the safety and efficacy of TH in burn management were included. No restrictions were placed on language or year of publication. Studies were eligible if they compared TH with a control group.

Studies were excluded if they were non-RCTs, including cohort studies, case reports, and case series. Additionally, animal studies, reviews, editorials, commentaries, meta-analyses, and research articles that did not report relevant outcomes were excluded. Studies without a comparison group were also not considered for analysis.

**Figure 1:**
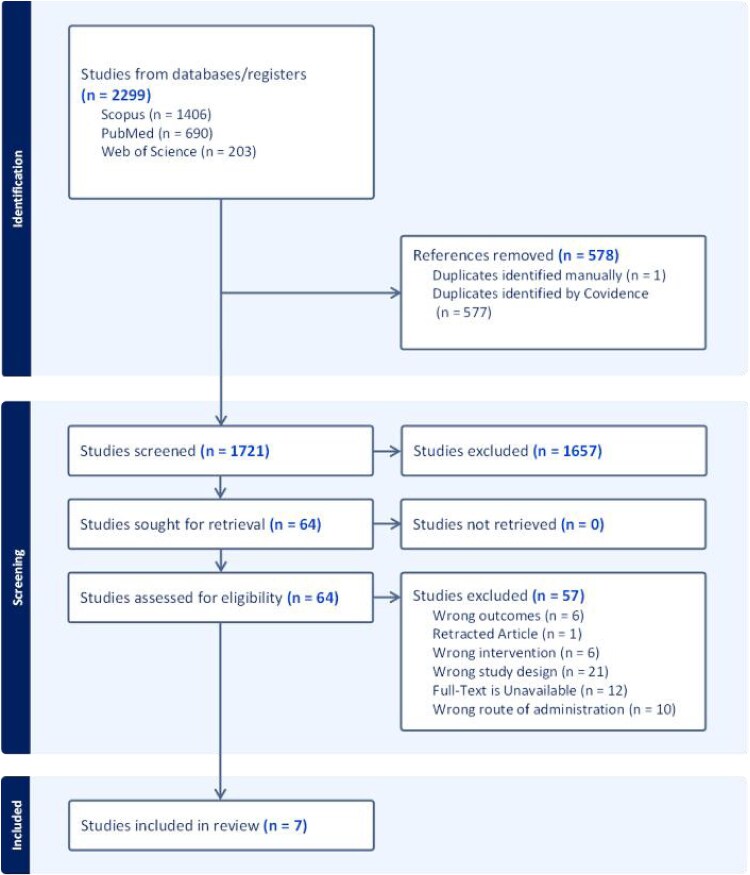
A PRISMA flowchart of the screening process

The screening process was conducted using the Covidence platform, a web-based tool designed to facilitate systematic reviews, and other types of research reviews.^10^ The study selection was conducted in two separate stages: (1) Title and abstract screening and (2) Full-text screening. Two independent reviewers screened the articles according to the criteria mentioned above, and a third reviewer was consulted in case of any dispute. Further details of the screening process are presented in Figure 1.

### Data extraction and quality assessment

Two reviewers independently extracted data from each study, and a third reviewer was consulted in case a consensus was not reached while resolving discrepancies.

The reviewers extracted the following baseline and outcome data from each qualifying study: The first author’s last name, year of publication, study design, sample size, and country of origin. Information on participant characteristics, including mean age (±standard deviation), percentage of female participants, and burn characteristics (cause, degree, and total burn surface area percentage), was collected. Details regarding the use of therapeutic adjuncts (TH adjuncts) were recorded. Outcomes assessed included analgesic drug usage rates, complication rates (such as bleeding and infections), pain scores, length of hospital stay, and mortality rates.

The quality assessment of RCTs was conducted using the Cochrane Risk of Bias 2 (RoB 2) tool. This tool provides a structured approach to evaluating potential biases that may affect trial findings. It assesses five key domains: (1) bias stemming from the randomization process, (2) bias resulting from deviations from intended interventions, (3) bias due to missing outcome data, (4) bias in outcome measurement, and (5) bias related to the selection of reported results.^11^ Two blinded evaluators independently conducted the risk of bias assessment. Any discrepancies between assessments were resolved through discussion.

### Statistical analysis

The R software version 4.4.1 was utilized for analysis.^12^ A double-armed, comparative meta-analysis compared the analgesic drug usage (1–2 times daily and 3–4 times daily), bleeding events, sepsis events, Visual Analog Scale (VAS) pain score, length of hospital stay, and mortality between the TH group and the control group without TH. Regarding the categorical variables (analgesic drug usage, bleeding, sepsis, mortality), a pooled risk ratio was computed alongside the 95% confidence interval. Regarding the continuous variables (VAS pain score and length of hospital stay), the mean difference was calculated alongside the 95% confidence interval. The fixed effects model with the Mantel–Haenszel method was used for all meta-analyses with insignificant heterogeneity. The cut-off value for significant heterogeneity was chosen to be an *I*^2^ value above 50%. Whenever significant heterogeneity was encountered, we used the random-effects model and the inverse-variance method for the analysis. Additionally, we conducted a leave-one-out test to reduce the heterogeneity when it was attributed to a single study. In all analyses, a *P*-value <.05 was considered significant.

## RESULTS

### Literature search

Upon applying our search strategy to three different databases, we yielded a total of 2299 results. Removal of duplicate entries kept 1721 studies for the title and abstract screening process. Sixty four studies were eligible for the full-text screening, out of which seven studies^13-19^ satisfied our inclusion criteria for the systematic literature review. Figure 1 presents a PRISMA flowchart of the screening process.

### Study characteristics

Overall, 503 burn cases were included in our meta-analytic review from seven studies. Hundred percent of the included studies involved randomization with at least one control group receiving non-TH treatment. All studies were limited to the African, Asian, and South American continents, with the majority being conducted in India (43%). There was evident variation in the age groups of the burn cases among the eight studies. Flame and scald burns were the most frequently reported burn etiologies. An in-depth breakdown of the patient characteristics is provided in Table 1.

**Table 1:** Baseline Characteristics

Study	Study design	Sample size	Country	Age in mean, SD	Control group	Burn cause	Burn degree	TH adjuncts	*N* (%) females	Burn %	Study conclusion
Venakatachalapathy 2007	Prospective randomized controlled trial	100	India	**Heparin group**: 44% from 15 to 25 years, 56% from 26 to 35 years **Control group**: 36% from 15 to 25 years, 64% from 26 to 35 years	Traditional routine: pain medications, intravenous resuscitation fluids, oral antibiotics, topical antimicrobial sulfur-base cream, water baths, debridement, tissue-releasing incisions, blood transfusions, and skin grafts	**Heparin group**: 90% flame, 10% scalds **Control group**: 80% flame, 20% scalds	Heparin group: 20% superficial second, 80% deep second Control group: 8% superficial second, 92% deep second	Pain medications, intravenous resuscitation fluids, oral antibiotics, water baths, debridement, tissue-releasing incisions, blood transfusions, and skin grafts	**Heparin group**: 21 (42%) **Control group**: 30 (60%)	**Heparin group**: 22% with a burn percentage of 5%-15%, 38% with a burn percentage of 16%-25%, 38% with a burn percentage of 26%-35%, 2% with a burn percentage of 36%-50% **Control group**: 18% with burn percentage 5%-15%, 28% with burn percentage 16%-25%, 28% with burn percentage 26%-35%, 26% with burn percentage 36%-50%	Less IV fluid requirements, less pain, lower requirements for pain medications, less antibiotic requirements, and lower costs in those treated with topical heparin compared to non-heparin-treated patients
Venakatachalapathy 2014	Prospective randomized controlled trial	100	India	**Heparin group**: 44% below 5 years, 56% from 6 to 15 years **Control group**: 36% below 5 years, 64% from 6 to 15 years	Traditional routine treatment of topical antimicrobial cream, debridement, and, when needed, skin grafts in the early postburn period	N/A	Heparin group: 70% superficial second, 30% deep second Control group: 60% superficial second, 40% deep second	Intravenous resuscitation fluids, oral antibiotics, water baths, debridement, tissue-releasing incisions, blood transfusions, and skin grafts	N/A	**Heparin group**: 20% with a burn percentage of 5%-15%, 30% with a burn percentage of 16%-25%, 32% with a burn percentage of 26%-35%, 18% with a burn percentage of 36%-50% **Control group**: 18% with burn percentage 5%-15%, 28% with burn percentage 16%-25%, 28% with burn percentage 26%-35%, 28% with burn percentage 36%-50%	Topical heparin in the first week after burn injury reduces LOS, IV fluid amounts, pain, need for dressings, and lowers the mortality rate in children
Amiruddin 2019	Prospective randomized controlled trial	60	Pakistan	10-60 years	30 patients were treated with conventional dressings with silver sulfadiazine, intravenous antibiotics, analgesics, and intravenous fluids	N/A	Second-degree burns covering 10%-60% of body surface area	Cleansing the wounds with heparin solution, routine blood tests, and not de-roofing the sores.	**Heparin group**: 15 (50%) **Control group**: 22 (73%)	**Heparin group**: 8% with a burn percentage of 10%-20%, 10% with a burn percentage of 21%-30%, 9% with a burn percentage of 31%-40%, 0% with a burn percentage of 41%-50%, 3% with a burn percentage of 51-60 **Control group**: 10% with burn percentage 10%-20%, 5% with burn percentage 21%-30%, 7% with burn percentage 31%-40%, 4% with burn percentage 41%-50%, 4% with burn percentage 51-60	Heparin decreases the utilization of analgesic drugs and reduces the duration of hospital stay
Barretto 2010	Prospective randomized controlled trial	58	Brazil	18 to 55 years	Conventional treatment with balneotherapy and silver sulfadiazine dressings under anesthesia	Fire or scald	Second- and third-degree burns	Daily hygiene care in bed and showers, morphine (0.05 mg/kg), dipyrone (7 mg/kg), paracetamol in case of fever	N/A	10%-30%	Topical heparin treatment showed higher analgesic effectiveness, fewer analgesic needs, and less pain compared with conventional treatment. It also resulted in less fever but more bleeding, without significant differences in infection rates or safety outcomes
Karagoz 2009	A randomized, triple-arm trial	45	Turkey	Mean age 24 years (range 3-55 years)	2 control groups: Scarfade (silicone gel), Epi-Derm (silicone gel sheet)	All except chemical burn	Patients with hypertrophic scars less than 6 months from injury	Topical onion extract including heparin and allantoin	20 females and 12 males Others (13) were not specified	N/A	While there was no significant difference between the Scarfade and Epi-Derm groups, both were significantly more effective than Contractubex. Silicone-based treatments, in gel or sheet form, proved superior to Contractubex for treating hypertrophic scars, suggesting that therapists should choose the most suitable treatment based on patient needs and scar characteristics
Manzoor 2019	Prospective randomized controlled trial	60	Pakistan	**Heparin group**: 28 ± 10 **Control group**: 27 ± 10	Polymyxin B sulfate, Bacitracin zinc for SPTB, silver Sulfadiazine cream for DPTB	**Heparin group**: 70% flame, 30% scald **Control group**: 80% flame, 20% scalds	19 individuals with superficial partial thickness burns, 41 individuals with deep partial thickness burns	All patients received intramuscular tetanus toxoid, Omeprazole 20 mg daily, and acyclovir 400 mg every 8 h. Analgesic (tramadol)	**Heparin group**: 16 (53.3%) **Control group**: 11 (36%)	**Heparin group**: Mean (SD): 14 (3) **Control group**: Mean (SD): 13 (3)	Applying topical heparin for PTB proved to be better at pain control and healing of wound when compared with conventional treatment
Patil 2019	Prospective randomized controlled trial	80	India	**Heparin group:** 45% from 15 to 25 years, 25% from 26 to 35 years, 20% from 36 to 45 years, 10% from 46 to 50 years **Control group:** 40% from 15 to 25 years, 25% from 26 to 35 years, 35% from 36 to 45 years, 0% from 46 to 50 years	40 treated with silver-based antimicrobial cream and paraffin gauze dressing	**Heparin group:** 0% chemical, 15% electrical, 35% flame, 50% scald **Control group:** 5% chemical, 15% electrical, 55% flame, 25% scald	Superficial to second-degree burns	Systemic antibiotics, analgesics, proton pump inhibitors, intravenous fluids	**Heparin group:** 22 (55%) **Control group:** 18 (45%)	**Heparin group:** 35% with burn percentage 5%-10%, 35% with burn percentage 11%-20%, 10% with burn percentage 21%-30%, 20% with burn percentage 31%-40% **Control group:** 15% with a burn percentage of 5%-10%, 25% with a burn percentage of 11%-20%, 25% with a burn percentage of 21%-30%, 35% with a burn percentage of 31%-40%	Topical heparin application decreases LOS, burn injury pain, and rates of wound infection. Topical heparin also reduces the time and cost needed for dressing

### Risk of bias assessment

As assessed by the RoB-2 tool, 100% of the included studies were graded as posing a high risk of bias overall. At the domain level, domain 4 (measurement of the outcome) was most frequently reported as having a high risk of bias. Conversely, all studies were assessed to pose a low risk of bias in domain 3 (missing outcome data). Six out of 7 studies were noted to have a high risk of bias arising from the randomization process, with a single study showing some concerns. Given that the number of included studies was fewer than ten, the risk of publication bias was not assessed.^20^ Further details are displayed in Supplementary Fig. 1.

### Analgesic drug usage

**Figure 2:**
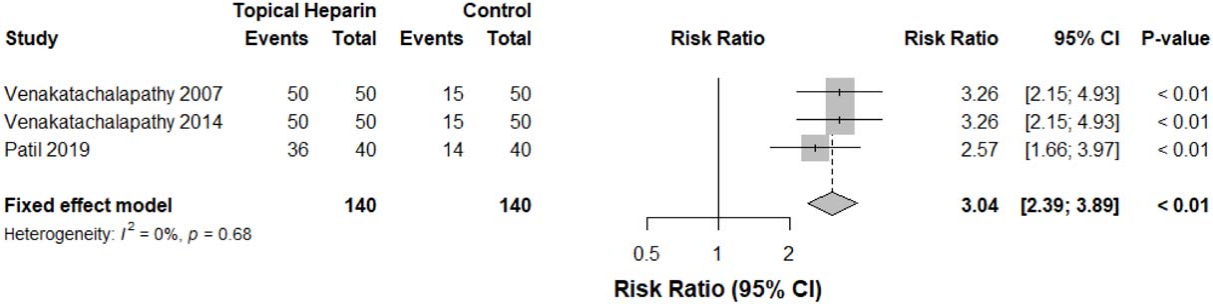
A forest plot comparing the rates of usage of analgesics 1 to 2 times daily

**Figure 3:**
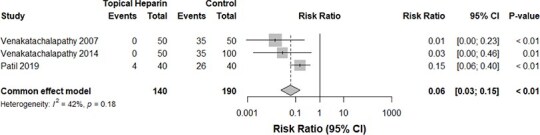
A forest plot comparing the rates of usage of analgesics 3 to 4 times daily

Three studies^13^^,^^14^^,^^19^ compared the relative rates of administration of analgesics between the TH and control groups. Our meta-analysis revealed an insignificant difference in the risk of analgesic administration 1 to 2 times daily between both groups (*P*-value: .68, *I*^2^ = 0%, 280 burn cases, RR for the TH group: 3.04, Figure 2). The administration of analgesics 3 to 4 times daily did not differ significantly between both groups, albeit with a trend toward lower administration events in the TH group (*P*-value: .18, *I*^2^ = 42%, 330 burn cases, RR for the TH group: 0.06, Figure 3).

### Complications and pain

**Figure 4:**
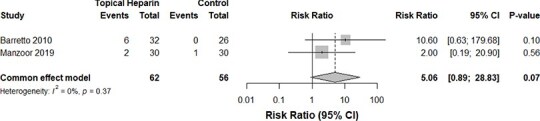
A comparison of bleeding events between the TH and control groups

**Figure 5:**
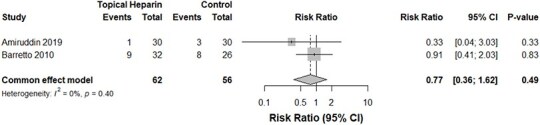
A forest plot demonstrating a comparison of sepsis rates between the TH and control groups

**Figure 6:**
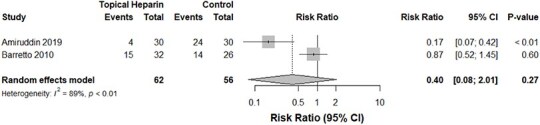
Infection rates in the TH groups vs the control group

Despite the TH group displaying a trend toward greater bleeding events, our pooled analysis of two studies^16^^,^^17^ concluded an insignificant difference between both groups (*P*-value: .37, *I*^2^ = 0%, 118 burn cases, RR for the TH group: 5.06, Figure 4). Similarly, those who received TH demonstrated no significant change in the risk of septicemia (*P*-value: .40, *I*^2^ = 0%, 118 burn cases, RR for the TH group: 0.77, Figure 5). The incidence of local wound infections in those who applied TH was significantly lower—by 60%—compared with the control group without TH. Heterogeneity, however, was significant in this analysis (*P*-value: <.01, *I*^2^ = 89%, 118 burn cases, RR for the TH group: 0.40, Figure 6).

**Figure 7:**
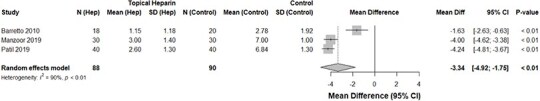
VAS pain score among those who received TH versus controls

**Figure 8:**
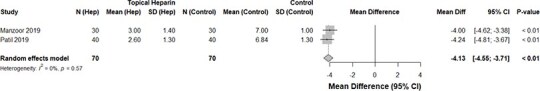
VAS pain score among those who received TH versus controls (Baretto 2010 excluded)

A comparison of the VAS pain scores between both groups yielded a significant reduction in pain experienced by the TH group (*P*-value: <.01, *I*^2^ = 90%, 178 burn cases, RR for the TH group: −3.34, Figure 7). Due to significant heterogeneity, we conducted a leave-one-out test which eliminated the significant reduction in pain skewed by the outlier Barretto et al.^17^ in the previous analysis (*P*-value: .57, *I*^2^ = 0%, 140 burn cases, RR for the TH group: −4.17, Figure 8).

### Length of hospital stay and mortalities

**Figure 9:**
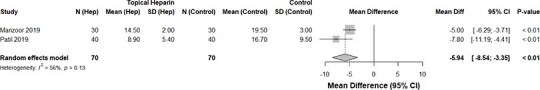
A forest plot comparing the length of stay between the TH and control groups

**Figure 10:**
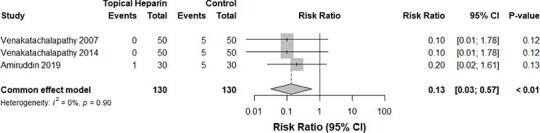
Mortality rates in the TH versus control group

Manzoor et al.^16^ and Patil et al.^14^ investigated the hospital stay durations in both groups; our meta-analysis of 140 patients from both studies indicated no significant reductions in the stay duration among those who received TH (*P*-value: .13, Figure 9). Similarly, fatality rates between both groups were comparable (*P*-value: .90, *I*^2^ = 0%, 260 burn cases, RR for the TH group: 0.13, Figure 10).

## DISCUSSION

This systematic review and meta-analysis comprehensively evaluated the safety and efficacy of TH in managing burn wounds by analyzing seven RCTs involving 503 patients. The findings offer valuable insights into pain relief, infection prevention, and general safety, yet highlight persistent concerns regarding the variability and quality of available evidence.

Our analysis demonstrated a significant reduction in VAS pain scores among patients treated with TH compared with controls (*P* < .01). This result is supported by various previous studies, confirming heparin’s potential analgesic properties through mechanisms such as anti-inflammatory activity, inhibition of inflammatory mediators, and improved local microcirculation.^21-24^ These biological effects might collectively decrease local edema and pain perception, thus enhancing patient comfort and facilitating wound management.^24^^,^^25^ While the reduction in pain is notable, the lack of a corresponding significant decrease in analgesic consumption between the TH and control groups warrants further investigation. Differences in analgesic prescribing practices, individual pain tolerance, and subjective pain assessments across studies may partly explain this discrepancy. For instance, Barretto et al. and Manzoor et al. reported significantly lower analgesic consumption among patients treated with TH.^16^^,^^17^ These variations, along with differing patient populations, could contribute to the differences in findings. Moreover, the reduction in pain, despite no significant change in analgesic use, aligns with the broader literature suggesting that TH’s anti-inflammatory properties, such as reducing local inflammation and enhancing microcirculation, play a role in pain relief and improved patient comfort.^7^

A notable outcome of our meta-analysis was the significant 60% reduction in local wound infections among patients treated with TH (*P* < .01). This finding aligns with several studies suggesting that heparin’s antimicrobial properties, such as inhibiting bacterial colonization, reducing biofilm formation, and enhancing tissue perfusion, are key mechanisms in preventing infections.^26^^,^^27^ Specifically, heparin has been shown to improve microvascular blood flow, which may hinder bacterial establishment in burn wounds.^7^^,^^27^ However, this encouraging result must be interpreted with caution due to substantial heterogeneity among the included studies (*I*^2^ = 89%). This variability may stem from differences in study methodologies, definitions of infection, types of burn injuries, and protocols for TH application. While previous research, such as the study by Cacique et al., has also reported favorable effects of heparin on infection rates, other studies have not found significant benefits.^23^ Conversely, studies like those by Biswas et al. and Barretto et al. reported no difference in infection outcomes between the treated and control groups.^17^^,^^22^

Concerning systemic complications, our study found no significant difference in the incidence of septicemia or bleeding events between the TH and control groups. Complementing our results, Manzoor et al. reported even lower complication rates associated with TH use. Together, these findings strongly support the excellent safety profile of TH in minimizing systemic risks, particularly noteworthy given its anticoagulant properties.^7^^,^^16^

Our findings revealed no statistically significant differences in hospital stay duration or mortality rates between the TH and control groups. These results are consistent with the findings of Vijayakumer et al., who similarly observed no effect of TH on these outcomes.^25^ While our findings differ from those of Biswas et al. and Masoud et al., who reported significant reductions in hospital stay with TH, it is important to note that these studies were based on smaller sample sizes,^22^^,^^28^ which may limit the generalizability of their conclusions.

Our meta-analysis builds on and extends the foundational work of Saliba et al. and Oremus et al. by narrowing the focus exclusively to TH in burn care and by incorporating newer RCTs. Saliba et al. provided a broad narrative survey of parenteral, topical, inhaled, and biomembrane preparations, highlighting heparin’s anti-inflammatory and microcirculatory benefits without quantitative synthesis.^7^ Similarly, Oremus et al. encompassed multiple administration routes with broader heterogeneity, and performed a mixed-route meta-analysis that suggested a mortality benefit (RR = 0.32).^8^ In contrast, our study delivers quantitative route-specific synthesis: we found a significant 60% reduction in local wound infections and a mean VAS pain score decrease of 3.34 points. Notably, unlike Oremus et al., we observed no mortality advantage, likely reflecting our topical-only inclusion criteria. Regarding safety outcomes, our study highlighted no significant increases in bleeding or sepsis thereby confirming the excellent tolerability of TH noted by Saliba et al. and later supported by Oremus et al.^7^^,^^8^ Finally, by synthesizing exclusively RCTs with standardized topical protocols, our review reduces the methodological heterogeneity that limited both reviews.

The clinical implications of our findings are multifaceted. While we did not observe a reduction in length of stay or mortality, the pain-relieving effects and the reduction in local wound infections suggest that TH can still be a valuable adjunctive therapy in burn care. Heparin’s ability to reduce local pain and infection could potentially enhance the comfort of burn patients and improve wound healing, making it a promising treatment for these challenges. Given its safety profile, with no significant increases in bleeding or septicemia, TH could be integrated into burn treatment regimens. However, clinicians should be aware of the variability across studies, and further trials with standardized protocols and larger sample sizes are needed to confirm these benefits.

### Limitations

The main limitation of this meta-analysis includes a high risk of bias, as all included studies had concerns regarding outcome measurement and randomization. In addition, there was high heterogeneity among studies in terms of VAS pain score and infection rates. Due to insufficient data, no subgroup analyses based on burn characteristics could be performed. Lastly, the limited generalizability of the results, due to the studies being conducted primarily in Africa, Asia, and South America—particularly India (43%)—raises concerns about their applicability to other populations, especially in Western healthcare settings.

### Strengths

This systematic review and meta-analysis offers the most comprehensive evaluation to date of the safety and efficacy of TH in burn management. A major strength of this study lies in its rigorous inclusion of RCTs, ensuring a higher level of evidence compared with previous reviews. The analysis encompasses multiple clinically relevant outcomes, including pain reduction, infection rates, hospital stay, and mortality, offering a well-rounded and detailed assessment of TH’s therapeutic potential.

## CONCLUSION

This study highlights the potential clinical relevance of TH as an adjunctive therapy in burn management. TH demonstrated significant reductions in pain and local wound infections, but no impact on mortality or hospital length of stay. Its favorable safety profile which is characterized by an absence of systemic adverse effects or increased bleeding risk, supports its use as a localized therapeutic agent. However, the limited scope of clinical benefit combined with substantial heterogeneity and methodological bias in existing studies, underscores the need for high-quality, standardized clinical trials with larger sample sizes. Indeed, TH shows promise as an adjunct for pain relief and infection prevention in burn patients, but its overall clinical impact is yet to be fully understood, necessitating more rigorous research to establish clearer guidelines for its use in burn care.

## Supplementary Material

Supplementary_Figure_1_iraf168

Supplementary_file_1_Search_Strategy_iraf168

## Data Availability

Data are available upon request to the corresponding author.

## Notes

Author contributions: Moussa Nassar (Conceptualization; Investigation; Writing—original draft; Methodology; Writing—review & editing; Validation; Supervision; Project administration; Data curation), Mohamed I. Mohamed (Writing—original draft; Methodology; Validation; Writing—review & editing; Formal analysis; Data curation; Supervision; Visualization), Maryam Shahid (Writing—original draft; Data curation), Rama Taha (Writing—original draft; Data curation), Rashed W. Alweshah (Writing—original draft; Data curation), Marwa R. Yousef (Writing—original draft; Data curation), Yousra Eltagouri (Writing—original draft; Data curation), Diaz G. Gustavo (Supervision; Validation; Conceptualization), Moussa Nassar (Conceptualization [equal], Data curation [equal], Investigation [equal], Methodology [equal], Project administration [lead], Validation [equal], Writing—original draft [equal], Writing—review & editing [equal]), Mohamed I. Mohamed (Data curation [equal], Formal analysis [lead], Methodology [equal], Validation [equal], Visualization [lead], Writing—original draft [equal], Writing—review & editing [equal]), Maryam Shahid (Data curation [equal], Writing—original draft [equal]), Rama Taha (Data curation [equal], Writing—original draft [equal]), Rashed W. Alweshah (Data curation [equal], Writing—original draft [equal]), Marwa R. Yousef (Data curation [equal], Writing—original draft [equal]), Yousra Eltagouri (Data curation [equal], Writing—original draft [supporting]), and Diaz G. Gustavo (Conceptualization [equal], Supervision [equal], Validation [equal]).

Moussa Nassar and Mohamed I. Mohamed contributed to this paper equally.

Funding: None declared.
