# Supplementary material for: Topical Heparin in Burns: A Systematic Review and Meta-Analysis of Randomized Controlled Studies
Source: J Burn Care Res. 2025 Aug 30;47(1):285–94. doi: 10.1093/jbcr/iraf168 (PMC12770970; doi:10.1093/jbcr/iraf168)
Supplement: Supplementary_Figure_1_iraf168 [file supplementary_figure_1_iraf168.pdf]

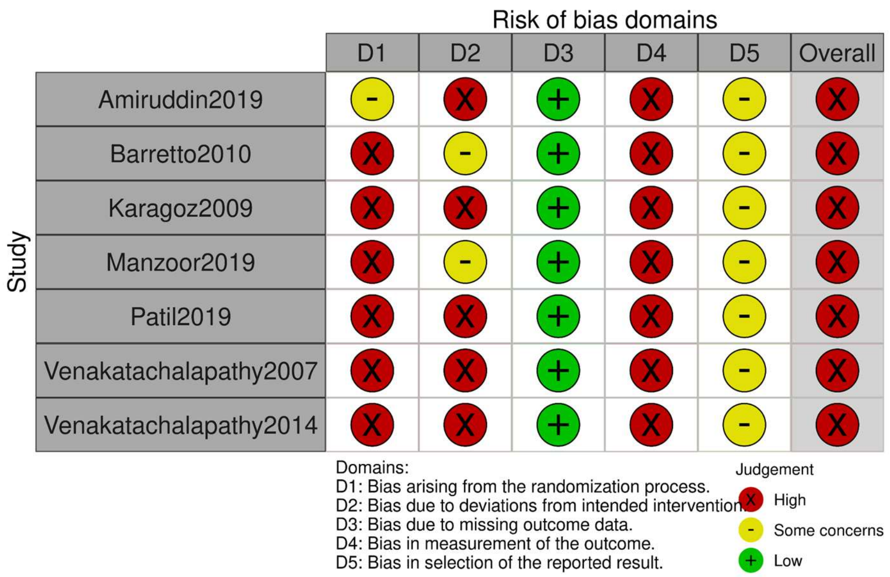
Risk of bias domains
D1
D2
D3
D4
D5
Overall
Study
Amiruddin2019
Barretto2010
Karagoz2009
Manzoor2019
Patil2019
Venakatachalapathy2007
Venakatachalapathy2014
Domains:
D1: Bias arising from the randomization process.
D2: Bias due to deviations from intended intervention.
D3: Bias due to missing outcome data.
D4: Bias in measurement of the outcome.
D5: Bias in selection of the reported result.
Judgement
High
Some concerns
Low

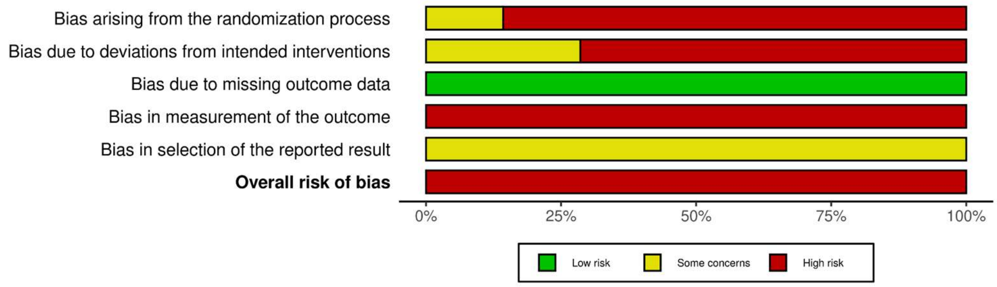
Bias arising from the randomization process
Bias due to deviations from intended interventions
Bias due to missing outcome data
Bias in measurement of the outcome
Bias in selection of the reported result
Overall risk of bias
0%
25%
50%
75%
100%
Low risk
Some concerns
High risk
