# Supplementary material for: Topical Heparin in Burns: A Systematic Review and Meta-Analysis of Randomized Controlled Studies
Source: J Burn Care Res. 2025 Aug 30;47(1):285–94. doi: 10.1093/jbcr/iraf168 (PMC12770970; doi:10.1093/jbcr/iraf168)
Supplement: Supplementary_file_1_Search_Strategy_iraf168 [file supplementary_file_1_search_strategy_iraf168.docx]

PubMed August 7, 2024 (690)
("Heparin" OR "topical heparin" OR "heparin therapy" OR "heparin application" OR "topical heparin treatment" OR "heparin treatment" OR "heparin administration") AND ("burns" OR "burn treatment" OR "burn wounds" OR "burn injuries" OR "burn management" OR "burn care" OR “burn*”).

Scopus August 7, 2024 (1406)
( TITLE-ABS-KEY-AUTH ( "Heparin" OR "topical heparin" OR "heparin therapy" OR "heparin application" OR "topical heparin treatment" OR "heparin treatment" OR "heparin administration" ) AND TITLE-ABS-KEY-AUTH ( "burns" OR "burn treatment" OR "burn wounds" OR "burn injuries" OR "burn management" OR "burn care" OR "burn*" ) )

Web of Science August 7, 2024 (203)
"Heparin" OR "topical heparin" OR "heparin therapy" OR "heparin application" OR "topical heparin treatment" OR "heparin treatment" OR "heparin administration" (Abstract) and "burns" OR "burn treatment" OR "burn wounds" OR "burn injuries" OR "burn management" OR "burn care" OR “burn*” (Abstract)
